# Biomarkers in neurodegenerative disorders: translating research into clinical practice

**DOI:** 10.3389/fnagi.2014.00281

**Published:** 2014-10-21

**Authors:** Manuel Menéndez-González

**Affiliations:** ^1^Neurology Unit, Hospital Álvarez-BuyllaOviedo, Spain; ^2^Departamento de Morfología y Biología Celular, Universidad de OviedoOviedo, Spain; ^3^Instituto de Neurociencias, Universidad de OviedoOviedo, Spain

**Keywords:** biomarker, MTAi, neurodegenerative disease, Neurodegenerative Diseases, Parkinson's Disease, Huntington's Disease, Frontotemporal dementia (FTD), CSF biomarkers

When I was invited by Frontiers to serve as Guest Editor of a Research Topic I had no doubts about the topic to address: the clinical use of biomarkers for neurodegenerative disorders. The prevalence of ND is increasing dramatically and one of the major challenges today is the need of early and accurate diagnosis, the other is the need of more effective therapies -in turn the development of such therapies also requires early and accurate diagnosis-. The main hope for an earlier and more accurate diagnosis comes from the use of biomarkers. Much research is being done trying to solve the many interrogates related to the role of biomarkers in the early diagnosis, differential diagnosis, and follow-up of neurodegenerative disorders. This is a filed where translational research is intense enough to make this topic interesting for basic researchers and clinicians. Indeed, the response to the call for contributions was very good. I had the opportunity to receive and edit a good amount of high quality articles devoted to diverse techniques across several ND from different perspectives.

Several articles are devoted to laboratory, biochemical biomarkers in neurodegenerative dementias. Researchers from Japan assessed the association of plasmatic annexin A5 with Alzheimer's disease and with Dementia with Lewy Bodies, and they found annexin A5 is indeed a good markers of both conditions (Sohma et al., 2013). Regarding the usefulness of CSF biomarkers in the differential diagnosis of FTLD vs. AD, an interesting review by D Irwin, J Trojanowski, and M Grossman highlight that CSF measurements of Aβ 1-42, t-tau, and p-tau differ significantly in FTLD from the abnormal levels seen in AD, and in a subset of both FTLD-tau and FTLD-TDP there are extremely low levels of t-tau of unclear etiology (Irwin et al., 2013). In other review, J Moreth, C Mavoungou, and K Schindowski discuss the role of CSF Aβ in ongoing clinical trials for AD as well as the latest regulatory strategies (Moreth et al., 2013). Researchers for the Alzheimer's Disease Neuroimaging Initiative (ADNI) evaluated the association of APOE with amyloid deposition, cerebrospinal fluid levels (CSF) of Aβ, tau, and p-tau, brain atrophy, cognition, and cognitive complaints in patients with early-Mild Cognitive Impairment (E-MCI) and cognitively healthy older adults (HC) and found that cortical amyloid deposition and CSF levels of Aβ were significantly associated with APOE ε 4 status but not E-MCI diagnosis, with ε 4 positive participants showing more amyloid deposition and lower levels of CSF Aβ than ε 4 negative participants (Risacher et al., 2013). These findings have practical repercussion, since clinicians, and researchers should consider APOE genotyping when evaluating biomarkers in early stages of the disease.

A number of articles addressed neuroimaging techniques. Researchers from Karolinska Institute and King's College London explored how the progression of atrophy in dementia may be predicted on the basis of the anatomical connectivity of the first atrophic region and found that the subcallosal medial prefrontal cortex is atrophied (in different extent depending on the brain hemisphere assessed) in FTD, SD, PNFA an AD (Lindberg et al., 2012). These results should also be taken into account when using neuroimaging biomarkers in the differential diagnosis of neurodegenerative dementias. There are two neuroimaging methods reports. One proposes the use of viscous fluid registration in voxel based morphometry studies to enhance sensitivity and localizing power in the software package SPM (Pereira et al., 2013) and the other describes the Medial Temporal Atrophy index (MTAi), a simple planimetric method for measuring the relative extent of atrophy of the Medial Temporal Lobe (MTA) in relation to the global brain atrophy (Menéndez-González et al., 2014a). Following with simple methods for assessing MTA in the clinical setting, researchers from Florida describe the utility of age-specific cut-offs for visual rating of medial temporal atrophy in classifying Alzheimer's disease, MCI, and cognitively normal elderly subjects (Duara et al., 2013).

Some other studies followed a multidimensional approach. In a multicentric study researchers performed a longitudinal and multidimensional assessment of individuals with the gene expansion for Huntington disease (HD) but not yet diagnosed who were evaluated annually. They identified three clusters that represented primarily cognitively impaired, behaviorally impaired, and cognitively preserved phenotypes. Thus, this multidimensional method results in an earlier diagnosis with less motor and cognitive impairment than a motor diagnosis. These findings have implications for designing preventive trials and providing clinical care in prodromal HD (Biglan et al., 2013). Again researchers for the ADNI, show how the short-term (1 year) prognosis of progression from amnesic MCI to dementia relates strongly to baseline markers of neurodegeneration, with the AD signature MRI biomarker of cortical thickness performing the best among MRI and CSF markers studied here. However, longer-term (3 year) prognosis in these individuals was better predicted by a marker indicative of brain amyloid. Prediction of time-to-event in a survival model was predicted by the combination of these biomarkers. These results provide further support for emerging models of the temporal relationship of pathophysiologic events in AD and demonstrate the utility of these biomarkers at the prodromal stage of the illness (Dickerson and Wolk, 2013). Gómez-Ramírez and Wu review the network-based approach in biomarker discovery as a source of key insights to fully understand the network degeneration hypothesis (the disease starts in specific network areas and progressively spreads to connected areas of the initial loci-networks, a concept also addressed in the article by Lindberg et cols.) and introduce a new framework for the quantitative study of biomarkers that can help shorten the transition between academic research and clinical diagnosis in AD (Gómez-Ramírez and Wu, 2014).

We also have two original reports on Parkinson's disease (PD) and parkinsonisms. One is on the use of DaTSPECT in the diagnosis of patients with hard-to-classify tremor who have a normal DaT-SPECT. Researchers from Spain and Mexico make a clinical follow up study and provide a list of the final diagnosis behind these cases (Menéndez-González et al., 2014b) at the time of discussing the role of DaTSPECT in the diagnosis of patients with tremor. In the other report, a group of researchers leaded by Prof Parnetti, assesses the differential role of CSF alpha-synuclein species, tau, and Aβ 42 in PD and conclude that the combination of CSF o/t-α-syn and Aβ 42/tau ratios improve the diagnostic accuracy of PD and that PD patients showing low CSF Aβ 42 levels at baseline are more prone to develop cognitive decline (Parnetti et al., 2014).

Finally, I also had the opportunity of publishing a couple of opinion articles: one on the many questions arising about the use of biomarkers for diagnosing neurodegenerative diseases routinely (Menéndez-González, 2014a) and other discussing the safety issues related to lumbar punctures performed for CSF analysis for the diagnosis of AD in daily clinical practice (Menéndez-González, 2014b).

Research on biomarkers on neurodegenerative disorders is a hot topic where much work has to be done yet. As we can learn from this Research Topic, biomarkers are allowing us to expand the knowledge on the biological and anatomical basis of neurodegenerative diseases and to implement diagnostic techniques in clinical practice and clinical trials.

## Conflict of interest statement

The author declares that the research was conducted in the absence of any commercial or financial relationships that could be construed as a potential conflict of interest.
